# Spontaneous loss versus stimulation gain in pump-probe microscopy: a proof of concept demonstration

**DOI:** 10.1117/1.JBO.25.3.036501

**Published:** 2020-03-13

**Authors:** Subir Das, Khalil Ur Rehman, Guan-Yu Zhuo, Fu-Jen Kao

**Affiliations:** a National Yang-Ming University, Institute of Biophotonics, Taipei, Taiwan; b China Medical University, Institute of New Drug Development, Taichung, Taiwan; c China Medical University Hospital, Integrative Stem Cell Center, Taichung, Taiwan

**Keywords:** nonlinear optics, pump-probe microscopy, stimulated emission, spontaneous loss, lock-in detection

## Abstract

**Significance:** The large background, narrow dynamic range, and detector saturation have been the common limiting factors in stimulated emission (SE)-based pump-probe microscopy, attributed to the very small signal overriding the very intense laser probe beam. To better differentiate the signal of interest from the background, lock-in detection is used to measure the fluorescence quenching, which is termed spontaneous loss (SL). The advantages are manifold. The spontaneous fluorescence signal can be well separated from both the pump and the probe beams with filters, thus eliminating the background, enlarging the dynamic range, and avoiding the saturation of the detector.

**Aim:** We propose and demonstrate an integrated pump-probe microscopy technique based on lock-in detection for background removal and dynamic range enhancement through SL detection.

**Approach:** The experimental setup is configured with a pulsed diode laser at a wavelength $\lambda_{\mathrm{pu}}=635\;\mathrm{nm}$, acting as a pump (excitation) and a mode-locked Ti:sapphire laser at a central wavelength $\lambda_{\mathrm{pr}}=780\;\mathrm{nm}$, serving as the probe beam (stimulation). Both pulse trains are temporally synchronized through high precision delay control by adjusting the length of the triggering cables. The pump and probe beams are alternatively modulated at different frequencies $f_{1}$ and $f_{2}$ to extract the stimulated gain (SG) and SL signal.

**Results:** SG signal shows saturation due to the irradiation of the intense probe beam onto the photodetector. However, the detector saturation does not occur at high probe beam power for SL detection. The fluorescence lifetime images are acquired with reduced background. The theoretical signal-to-noise ratios for SG and SL are also estimated by photon statistics.

**Conclusion:** We have confirmed that the detection of SL allows the elimination of the large background without photodetector saturation, which commonly exists in SG configuration. This modality would allow unprecedented manipulation and investigation of fluorophores in fluorescence imaging.

## 1 Introduction

Pump-probe microscopy has been a versatile and powerful platform that takes advantage of transient absorption (TA) and gain in nonlinear optical processes with many imaging modalities, including both labeled and label-free ones. The labeled imaging modalities, such as stimulated emission (SE), excited state absorption, and ground state depletion, are able to reveal molecular specificity, improve resolution, and enhance penetration depth.^1^^–^^5^ The label-free ones include stimulated Raman scattering (SRS)^6^ and TA.^7^ These imaging modalities have been shown to reveal the structural features and transient phenomena in biology and chemistry at picosecond or femtosecond time scales.^8^^–^^12^ In the pump-probe technique, the pump pulse is used to excite the sample and the induced changes are then monitored by the synchronized probe pulse. Notably, lock-in detection is used throughout the pump-probe microscopy for both labeled and label-free imaging to recover the relatively small modulated signals from the very large background.

Among the above-mentioned modalities, SE is one of the most versatile techniques for scanning optical microscopy, with its renowned application in stimulated emission depletion (STED) microscopy to allow spatial resolution far beyond the diffraction limit.^13^ In STED microscopy, a common approach is to excite the electrons from ground states to excited states and another laser beam at a wavelength that partially overlaps the emission spectrum of the fluorophore is then used to turn the excited fluorophores to a nonfluorescent state (dark state) by SE. In this way, SE increases the number of photons (gain) in the probe beam (stimulated gain, SG), while it also quenches the fluorescence emission process, known as spontaneous loss (SL). In this case, the rate equations for populations of the ground state $\left(S_{0}\right)$ and of the excited state $\left(S_{1}\right)$ can be written as^14^

$$\frac{dS_{0}}{dt}=-k_{\mathrm{exc}}S_{0}+(k_{\mathrm{fl}}+k_{\mathrm{STED}})S_{1},$$ (1)

$$\frac{dS_{1}}{dt}=k_{\mathrm{exc}}S_{0}-(k_{\mathrm{fl}}+k_{\mathrm{STED}})S_{1},$$ (2)

where $k_{\mathrm{exc}}=\sigma_{\mathrm{abs}}I_{\mathrm{exc}}$, $k_{\mathrm{fl}}=1/\tau$, and $k_{\mathrm{STED}}=\sigma_{\mathrm{STED}}I_{\mathrm{STED}}$ are the rate of excitation caused by the pump beam, rate of spontaneous emission, and rate of SE caused by the STED beam, respectively. Therefore, increasing the STED beam would shorten the excited state lifetime, $\tau =\frac{1}{k_{\mathrm{fl}}+k_{\mathrm{STED}}}$. This feature of STED with a low intensity STED beam has been used to improve image resolution.^14^ Note that the transitions of SE take place in both real states (fluorescence) and virtual states (SRS). In SRS, the pump and Stokes beams are illuminated on the sample when the frequency difference between the pump and the Stokes matches the specific molecular vibrational frequency of a chemical bond. As a result, the Stokes beam experiences photon gain (stimulated Raman gain). On the other hand, the pump beam experiences photon loss (stimulated Raman loss).

In addition, SE-based pump-probe microscopy was carried out for undetectable fluorophores detection,^15^ subdiffraction fluorescence lifetime imaging,^16^ and background-free fluorescence imaging.^17^ SE is also a two-photon process working through real state transition, which has an equivalent cross section several orders of magnitude greater than the virtual ones. Note that in scanning optical microscopy, the penetration depth and signal-to-background ratio are two key advantages claimed by two-photon (2P) excitation due to the nonlinear intensity dependence of the absorption. SE reduced fluorescence microscopy was demonstrated to extend the fundamental depth limit of 2P fluorescence imaging.^18^ A femtosecond laser is often required to achieve effective excitation efficiency since the transition is through virtual states, which renders such an imaging system being costly, bulky, and complex. In comparison, SE could realize the 2P process with the use of gain-switched laser diodes, greatly reducing the cost and the complexity of operating a femtosecond laser.

In this paper, we are presenting an integrated pump-probe microscopy setup for the detection of both the SG and the SL. Critically, SL detection allows the reduction of high background in the signal and more flexibility in selecting detectors and the corresponding electronics for signal processing. A comparison between SG and SL is highlighted in Table 1.

**Table 1:** Basic differences between SG and SL.

Comparison	SG	SL
Characteristics	Forward emission	4-π (epi) emission
Background and noise	Large background from the probe laser	Laser background free with minute shot noise from spontaneous emission
Detection technique	Heterodyne technique: the modulation transferred signal to the probe beam is extracted by lock-in amplifier	Both heterodyne and gated photon counting [with use time-correlated single-photon counting (TCSPC)] can be used
Working range	Limited by the saturation level of the photodetector	Limited by the dynamic range of the detector
Amplification and gain	Usually there is no gain for the detector used	Amplification is allowed

## 2 Experimental

### 2.1 Working Principle of Lock-In Detection for Signal Extraction from Stimulated Gain and Spontaneous Loss

**Fig. 1:**
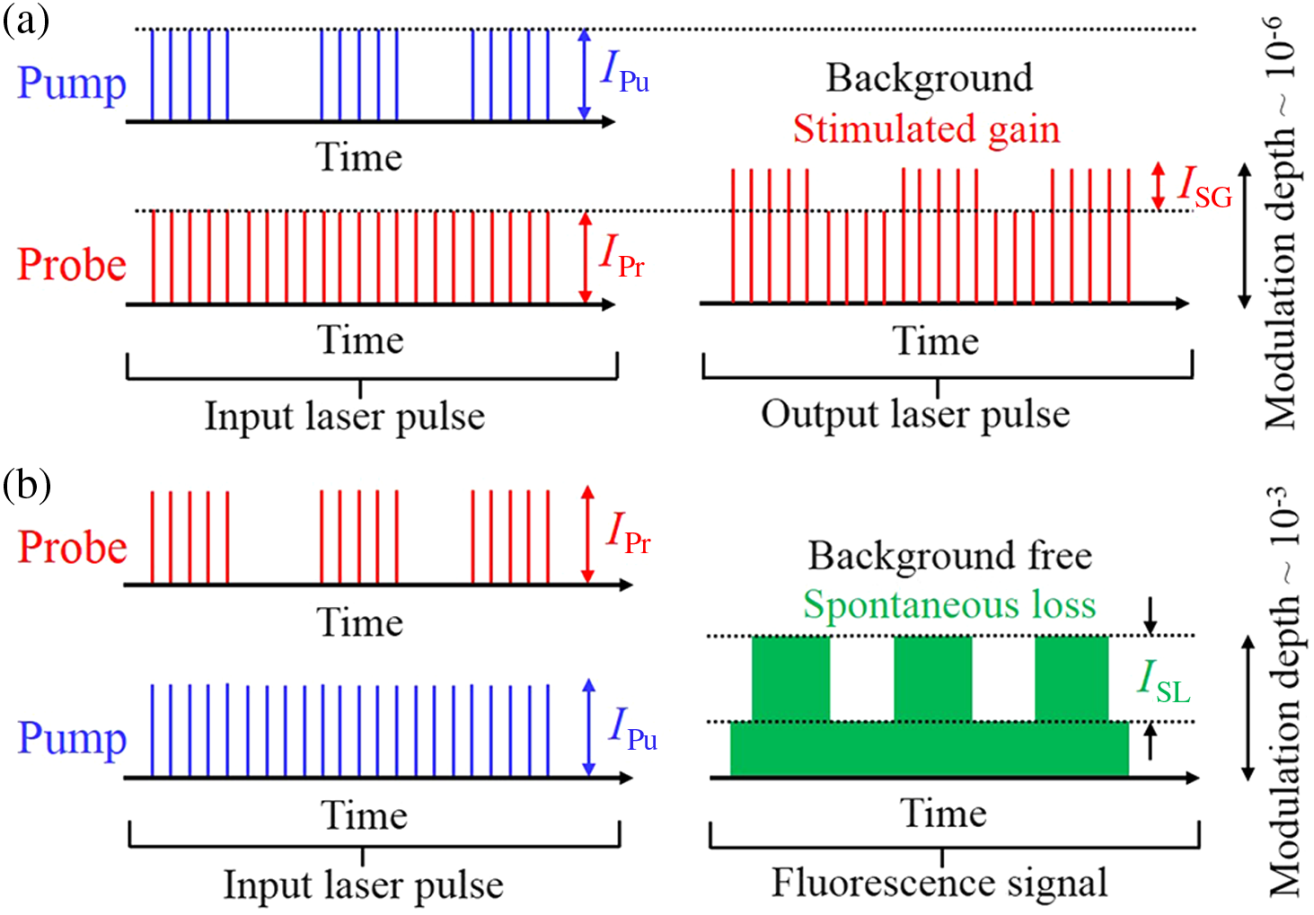
Working principle of modulation transfer for (a) SG and (b) SL in pump-probe microscopy. Either the pump beam or the probe beam is modulated for SG or SL detection. The SG ($I_{\mathrm{SG}}$) and SL ($I_{\mathrm{SL}}$) signals can be extracted by demodulating the probe ($I_{\mathrm{Pr}}$) beam and the spontaneous emission accordingly.

The working principle of modulation transfer that carries the SE signal in pump-probe microscopy for SG and SL is shown in Fig. 1. The pump and the probe beams are alternatively modulated at a selected frequency, f, to extract SG and SL, respectively.

For SG detection, the sample is irradiated with the modulated pump beam and SG is then detected by demodulating the probe beam at the same frequency by a lock-in amplifier. In the same way, when the sample is excited with the unmodulated pump and the modulated probe beam, the loss in spontaneous emission due to SE is extracted by demodulation with the modulation frequency (on the probe beam).

### 2.2 Spectral Detection Scheme for Both Stimulated Gain and Spontaneous Loss

**Fig. 2:**
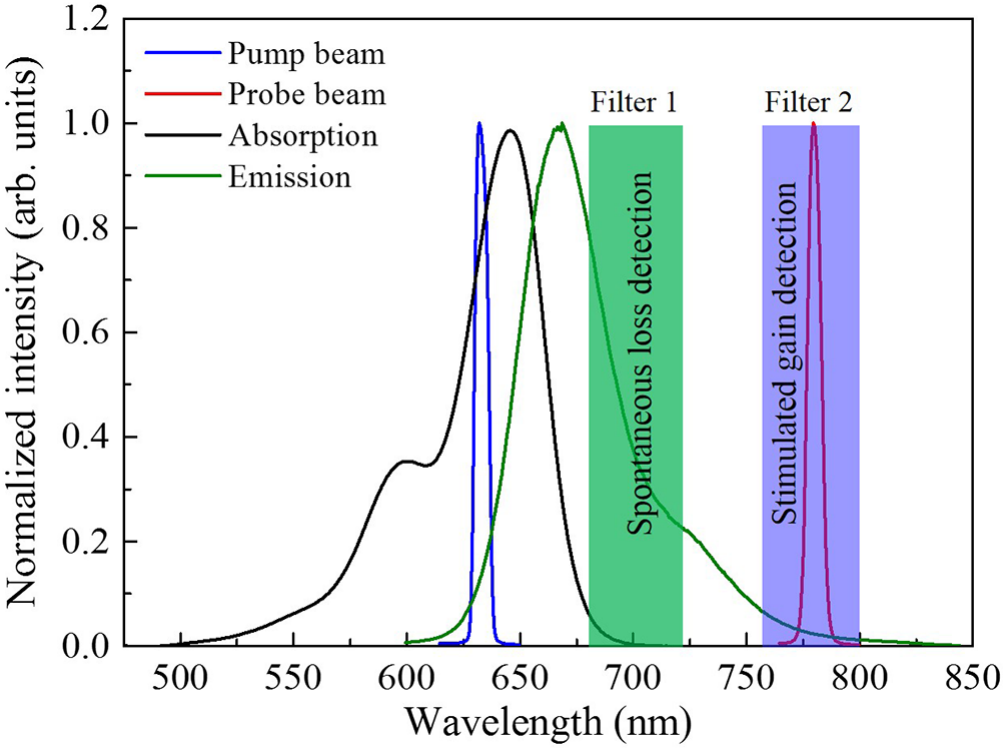
Spectral distribution of the absorption and emission spectrum of ATTO 647N dye and the laser (pump and probe) beams. The SG and SL signals are separately detected at two different channels using appropriate band pass filters.

The versatile and robust ATTO 647N fluorescent dye (ATTO 647N, ATTO-TEC, Germany) is used for demonstration. The absorption and fluorescence emission spectra of the red fluorescent dye along with the pump (excitation) and probe (stimulated) laser beams are shown in Fig. 2. The pump ($\lambda_{\mathrm{pu}}=635\;\mathrm{nm}$) and the probe ($\lambda_{\mathrm{pr}}=780\;\mathrm{nm}$) beams are selected at two different wavelengths that match well with the absorption and emission band spectra of the dye. The spectral filter sets are used in the experiment for two functions: (i) blocking the pump beam completely and allowing the probe beam only that carries SE signal and (ii) blocking both the pump and the probe beams and allowing only fluorescence emission. Critically, in the detection of SL, a band pass filter (marked by green) is used to reject both the pump and the probe wavelengths and pass only the spontaneous emission. While for the detection of SG, a band pass filter (marked by blue) is used to block the pump beam completely and pass the probe beam along with the florescence emission (which is a substantial source of background in SG detection).

### 2.3 Optical Microscope Setup

**Fig. 3:**
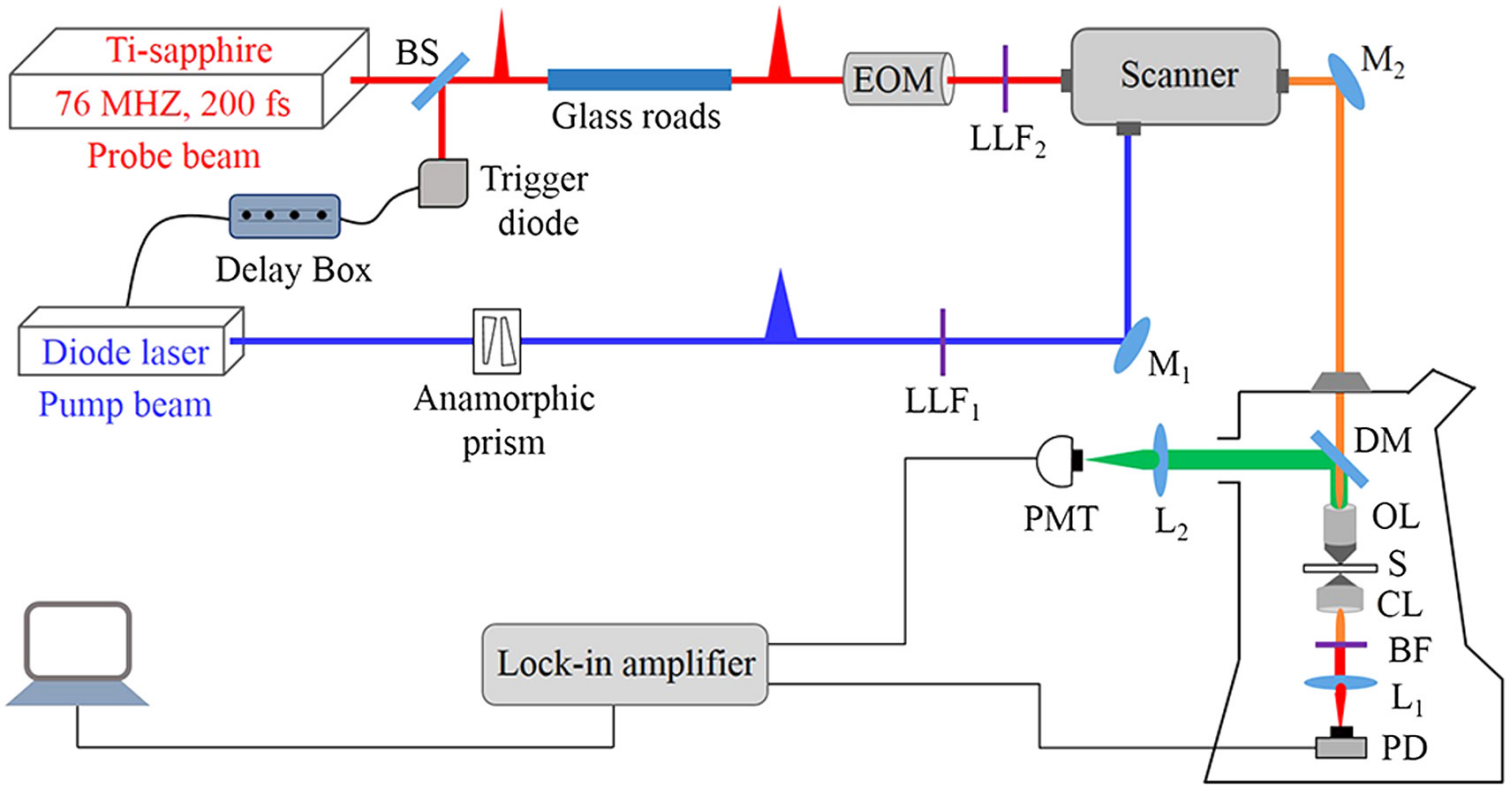
Experimental setup of the pump-probe microscopy for the SG and the SL. EOM, electro-optic modulator; LLF, laser line filter; M, mirror; DM, dichroic mirror; BS, beam splitter; OL, objective lens; S, sample; CL, condenser lens; L, lens; F, bandpass filter; PD, photodiode; PMT, photomultiplier tube. The backscattered scheme is used to detect the SL whereas the SG is detected in transmission mode.

The schematic of the experimental setup for the SG and the SL is shown in Fig. 3. Our pump-probe microscope is configured with a pulse diode laser (LDH-D-C-635M, PicoQuant, Germany) with the pump (excitation) beam at a wavelength of $\lambda_{\mathrm{pu}}=635\;\mathrm{nm}$ and a pulse width of ∼120  ps, which is synchronized with the mode-locked Ti-sapphire laser (Mira F-900, Coherent Inc.), serving as the probe beam operated at a central wavelength of $\lambda_{\mathrm{pr}}=780\;\mathrm{nm}$ through a trigger diode (TDA200A, PicoQuant, Germany). The maximum pump and probe beams power are set at 2.6 and 50 mW accordingly. The time delay (τ) between the pump and probe pulses are precisely controlled by adjusting the length of the triggering cable and setting the nanosecond delay box (Ortec 425A, Ametek). Two laser line filters (FL635-10 and FL780-10, Thorlabs) are used to remove the unwanted wavelengths that are associated with the pump and probe beams. The probe beam pulses (200 *fs*) are passed through two 15-cm long dispersive glass rods (SF-6) for pulse width stretch (to ∼2.6  ps) to avoid 2P excitation. An anamorphic prism (PS875-A, Thorlabs) is used to transform the elliptical mode of the pump beam into a circular one for better mode matching and tighter focusing. Both beams are coupled into a laser scanning unit (FV300, Olympus, Japan) through a dichroic mirror (FF01-720/SP, Semrock). The combined beams are focused onto the sample by an objective lens (UPlanFL 10X 0.30, Olympus, Japan) and the transmitted light is collected by another objective lens (UPlanFL 10X 0.30, Olympus, Japan). For SG, the pump beam is modulated with a lock-in amplifier (HF2LI, Zurich Instrument, Switzerland) at the frequency of 100 KHz. A bandpass filter (FF01-769/41-25, Semrock) is used to block the pump beam completely and let only the probe beam along with some fluorescence to pass through the filter. The SE photons gained by the probe beam propagating in the transmission direction are detected by a silicon photodiode (PDA36A, Thorlabs). For comparison, in SL, the probe beam is modulated with an electro-optic modulator (M350-80LA, Conoptics Inc.) also at the frequency of 100 KHz. The spontaneous emission is reflected in the backscattered direction by a dichroic mirror (ZT685dcrb, Chroma Technology). A bandpass filter (FF01-700/13-25, Semrock) is placed before the photomultiplier tube (PMT) to completely block the pump and the probe beams. The SL signal is detected by demodulating the output of the PMT (R376, Hamamatsu, Japan) with the lock-in amplifier. The time constant of the lock-in amplifier is set at 2 ms. The output of the lock-in amplifier is connected to the analog-to-digital channel of the scanning unit to reconstruct the images. For SG and SL signals’ measurements, the fluorescent ATTO 647N dye is dissolved in deionized water with various concentrations (0.1 to 1 mM). The dye solution is injected into the microchannel slide (15μ-slide, ibidi GmbH, Germany) for testing. A piece of lens cleaning tissue paper is immersed inside the ATTO 647N dye solution and sandwiched between two cover glass slides for time-resolved imaging.

## 3 Results and Discussions

**Fig. 4:**
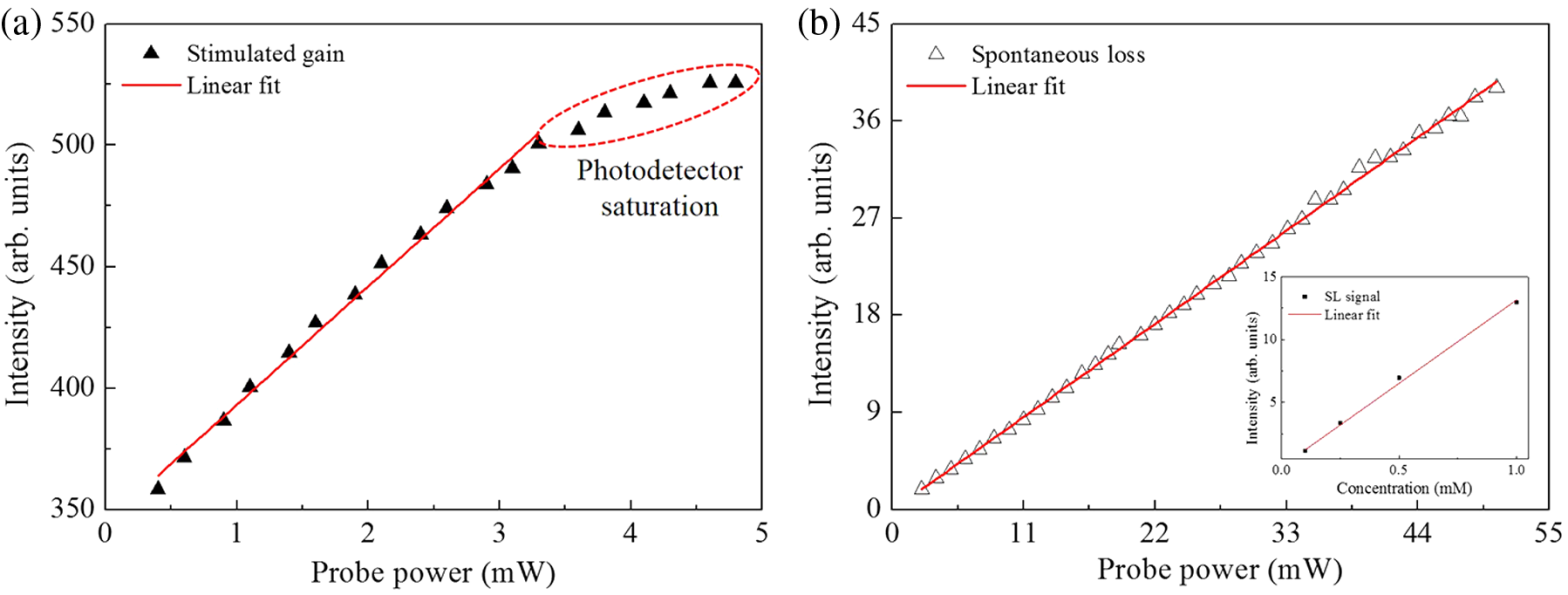
The (a) SG and (b) SL signals as a function of the probe beam power. The photodetector saturation effect is marked with a red dashed oval. The pump beam power is fixed at 2.6 mW. Inset: SL signal as a function of various dye concentrations (0.1 to 1 mM).

Figure 4(a) shows the SG signal as a function of the probe beam power. When the probe laser power reaches 3.5 mW, the SG signal starts to show saturation due to the intense power of the probe beam on the photodetector.

In such a high NA setting, the SG contains a large background, which is attributed to the spontaneous emission caused by the pump beam. Notably, the SE is also the dominant fluorescence quenching process with the wavelength-dependent SE cross section. ^19^ The SG and the SL are thus correlated with each other. The fluorescence reduction rate of a single molecule can be described as

$$R_{\mathrm{SERF}}=R-R^{\prime }=f_{\mathrm{rep}}k_{\mathrm{exc}}\tau_{\mathrm{exc}}\eta \frac{k_{\mathrm{SE}}}{k_{\mathrm{fl}}+k_{\mathrm{SE}}},$$ (3)

where $R_{\mathrm{SERF}}$, $f_{\mathrm{rep}}$, $k_{\mathrm{exc}}$, $\tau_{\mathrm{exc}}$, η, $k_{\mathrm{SE}}$, and $k_{\mathrm{fl}}$ are the fluorescence reduction rate, repetition rate, excitation rate, pulse width, fluorescence quantum yield, SE rate, and fluorescence emission rate, respectively.^18^ In SG measurements, some spontaneous fluorescence signal (forming the background) is always present along with the SE signal and cannot be filtered out since the probe beam lies within the emission band of the fluorescent dye. For comparison, Fig. 4(b) shows the SL signal increases linearly with the probe beam power. The SL signal does not saturate at a high laser power of ∼50  mW, and the detected SL dose not contribute to any background or detector saturation.

**Fig. 5:**
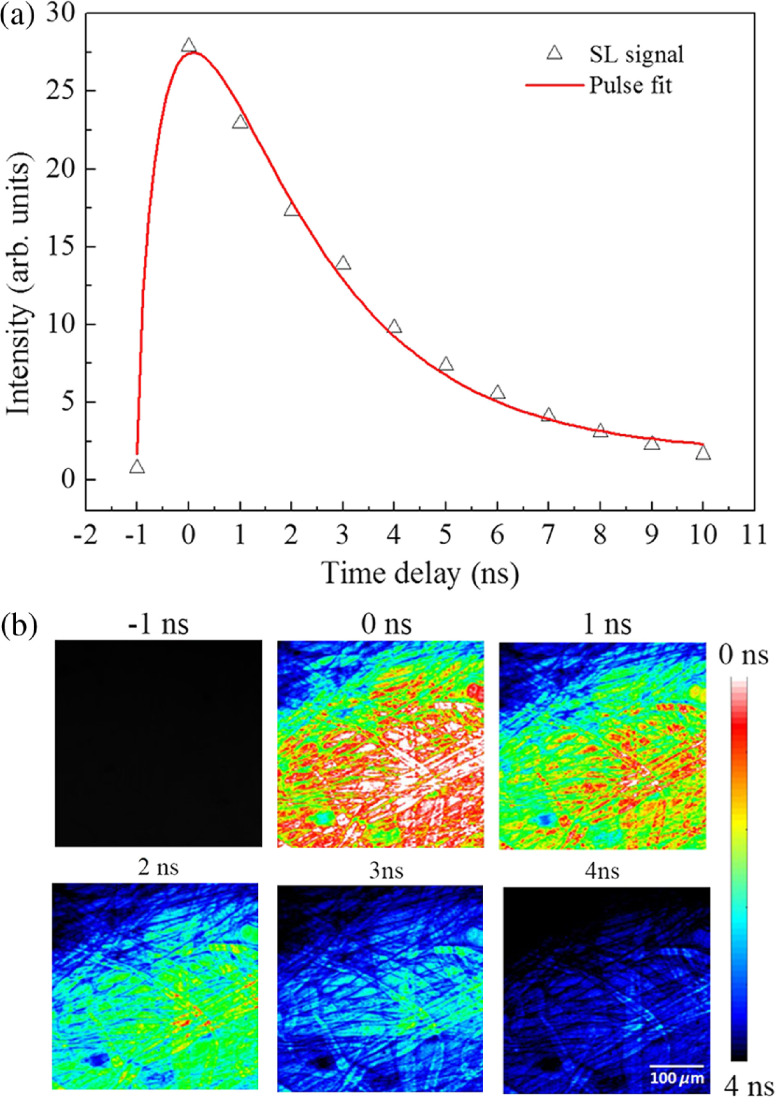
(a) SL signal as a function of the relative time delay between the pump and the probe pulses. (b) The fluorescence lifetime images of lens cleaning tissue paper at different delay times. Note that all the color coded images are acquired at 512×512  pixels with a scale bar of 100  μm.

The SL signal as a function of time delay between two pulses is shown in Fig. 5(a). In a fluorescence quenching experiment, the fluorescence intensity and relative delay are given as

$$\frac{I_{0}-I}{I}=q\;\exp(-t_{d}/\tau ),$$ (4)

where $I_{0}$ and I are the intensities in the absence and presence of SE pulses, q is the extent of quenching, $t_{d}$ is the relative time delay between the pump and probe pulses, and τ is the fluorescence lifetime of the fluorophore.^20^ The fluorescence lifetime images with different delay times are shown in Fig. 5(b). When compared with the data acquired in our previous work,^21^ the lifetime images are obtained with greatly reduced backgrounds.

**Fig. 6:**
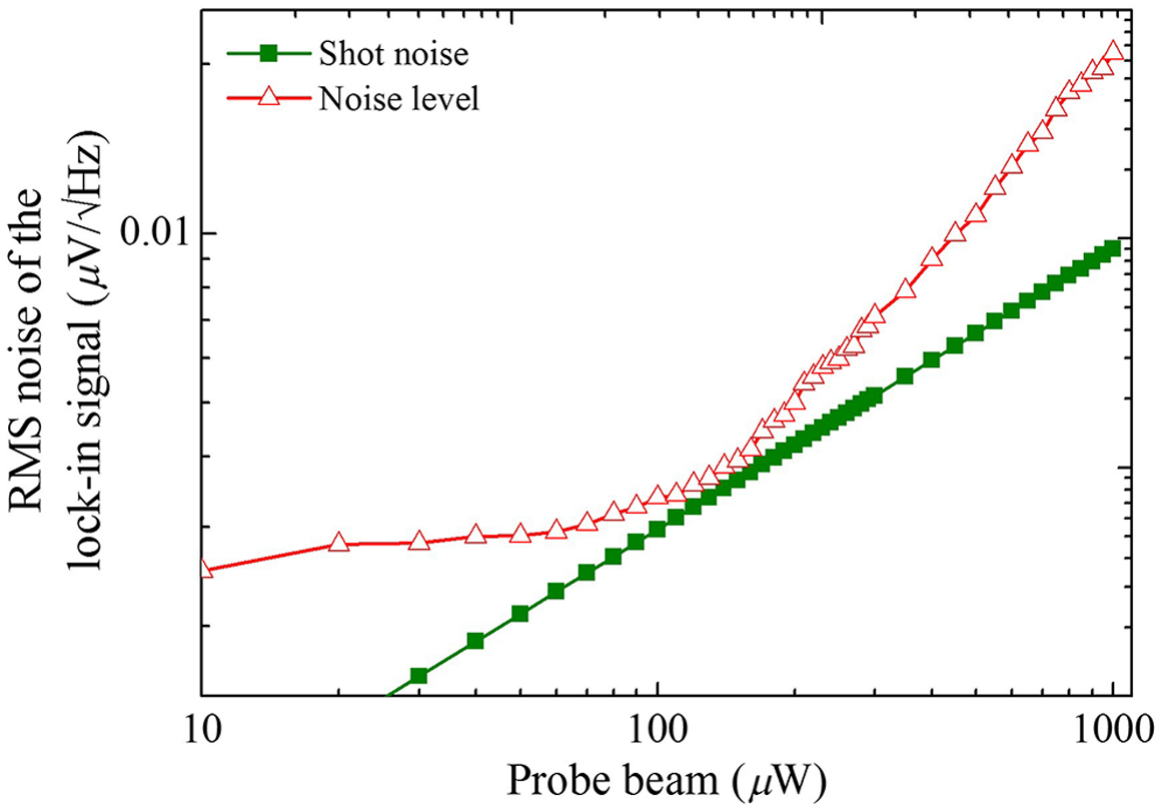
Noise level of the lock-in signal as a function of optical power of the probe beam. The triangle (red) and square (green) represent the measured noise level and theoretical shot noise, respectively.

In SG detection, the probe beam carrying the SE signal directly irradiates the photodetector and contributes substantial noise due to its high intensity and fluctuation. The noise level due to the probe beam is evaluated by switching off the pump beam. The root-mean-square noise, which is the standard deviation of the detected signal over a period of time, is measured as the input power of the probe beam, as shown in Fig. 6. The experimental result is compared with the theoretical shot noise level, which is evaluated by Eq. (5) with the following parameters for the photodetector: gain, $G=0.75\times 10^{3}\;V/A$, elementary charge, $q=1.6\times 10^{-19}\;C$, responsivity (r)=0.49  A/W at 780 nm, and optical power, P.

$$v_{\text{shot}}=G\sqrt{2qrP}.$$ (5)

In this condition, the shot noise level is limited at a very low optical probe power (∼110 to 160  μW). As the power of the probe beam increases, the main source of noise is switched to the laser noise. However, the laser intensity noise does not affect the SL signal except for shot noise.

In an optical imaging system, signal-to-noise ratio (SNR) is a critical parameter, which is also estimated by theoretical calculation. Here, we used Poisson statistics to evaluate the theoretical SNRs of the SG and SL. The experimental parameters for theoretical calculation are listed in Table 2.

**Table 2:** Experimental parameters for theoretical SNR calculation.

Parameter	Value
Laser repetition frequency $\left(f_{\mathrm{rep}}\right)$	76 MHz
Pump wavelength $\left(\lambda_{\mathrm{pu}}\right)$	635 nm
Probe wavelength $\left(\lambda_{\mathrm{pr}}\right)$	780 nm
Pump power $\left(P_{\mathrm{pu}}\right)$	2.6 mW
Probe power $\left(P_{\mathrm{pr}}\right)$	3.6 mW
Pump photons per pulse ($n_{\mathrm{pu}/\text{pulse}}$)	$1.09\times 10^{8}$
Pump airy disc diameter $\left(d_{\mathrm{pu}}\right)$	$2.58\times 10^{-6}\;m$
Pump axial resolution $\left(z_{\mathrm{pu}}\right)$	$9.38\times 10^{-6}\;m$
Pump beam waist area $\left(A_{\mathrm{pu}}\right)$	$5.23\times 10^{-12}\;m^{2}$
Probe photons per pulse ($n_{\mathrm{pr}/\text{pulse}}$)	$1.86\times 10^{8}$
Probe airy disc diameter $\left(d_{\mathrm{pr}}\right)$	$3.17\times 10^{-6}\;m$
Probe axial resolution $\left(z_{\mathrm{pr}}\right)$	$1.15\times 10^{-5}\;m$
Probe beam waist area $\left(A_{\mathrm{pr}}\right)$	$7.89\times 10^{-12}\;m^{2}$
Concentration (C)	1 mM
Avogadro’s number $\left(N_{A}\right)$	$6.02\times 10^{23}\;{\mathrm{mol}}^{-1}$
Speed of light (c)	$3\times 10^{8}\;m/s$
Planck’s constant (h)	$6.626\times 10^{-34}\;\mathrm{Js}$
Numerical aperture (N.A)	0.3
Bandpass filter efficiency	23%
Refractive index (η)	1.33
Quantum efficiency of ATTO 647N $\left(\eta_{\mathrm{fl}}\right)$	65%
Absorption cross-section $\left(\sigma_{\mathrm{abs}}\right)$	$2.5\times 10^{-20}\;m^{2}$
SE cross-section $\left(\sigma_{S.E.}\right)$	$5\times 10^{-21}\;m^{2}$
Lifetime of $\left(\tau_{\mathrm{fl}}\right)$	3.5 ns
Probability of SE $\left(\frac{k_{S.E.}}{k_{\mathrm{fl}}+k_{S.E.}}\right)$	0.058
Solid angle at N.A = 0.3 (Ω)	0.0128
No. of excited molecules per area ($CN_{A}z_{\mathrm{pu}}$)	$5.64\times 10^{18}\;m^{-2}$
No. of stimulated molecules per area ($CN_{A}z_{\mathrm{pu}}\frac{n_{\mathrm{pu}/\text{pulse}}}{A_{\mathrm{pu}}}\sigma_{\mathrm{abs}}$)	$2.93\times 10^{18}\;m^{-2}$

The number of spontaneous photons per pulse in the pump beam waist is estimated as

$$(\text{No. of excited molecules}/\text{area})\times (n_{\mathrm{pu}/\text{pulse}})\times \sigma_{\mathrm{abs}}\times \Omega \times \eta_{\mathrm{fl}}=1.27\times 10^{5}.$$

Similarly, the number of SE photons per pulse in the probe beam waist is then

$$(\text{No. of stimulated molecules}/\text{area})\times \sigma_{S.E.}\times (n_{\mathrm{pr}/\text{pulse}})\times (\frac{k_{S.E.}}{k_{\mathrm{fl}}+k_{S.E.}})\times \text{filter efficiency}\;=3.63\times 10^{4}.$$

The SNR of SG can be estimated as

$$\mathrm{SNR}=\frac{\mathrm{SE}\text{ signal}}{\sqrt{\left\{(SE\text{ signal})+(\text{Spontaneous emission})\right\}}},\;\mathrm{SNR}=\frac{\left(3.63\times 10^{4}\right)}{\sqrt{\left\{(3.63\times 10^{4})+(1.27\times 10^{5})\right\}}},\;\mathrm{SNR}=89.$$

The SNR of SG is limited by the photodetector saturation window due to the strong probe beam power.

The SNR of SL can be calculated as

$$\mathrm{SNR}=\frac{\text{Spontaneous signal}}{\sqrt{\text{Sponatenous signal}}},\;\mathrm{SNR}=\frac{\left(1.27\times 10^{5}\right)}{\sqrt{\left(1.27\times 10^{5}\right)}},\;\mathrm{SNR}=356.$$

In SL, the selection of a high gain detector, such as a PMT, would improve the SNR.

As discussed above, the benefits and limitations of SL are illustrated using conventional analog lock-in detection mode. Notably, the analog measurements are always affected by the detector’s gain [such as a photodiode (PD) and PMT] and the electronics noise (such as thermal noise, flicker noise, and shot noise). For a sine wave modulation, the effective count rate $\left(r_{T}\right)$ over a bin time (T) can be expressed as^22^

$$r_{T}(t)=r_{0}+A[\frac{\sin(\pi f_{m}T)}{\pi f_{m}T}]\sin(2\pi f_{m}t+\pi f_{m}T+\theta ),$$ (6)

where $r_{0}$ is a constant rate, A is the modulation depth, and $f_{m}$ is the modulation frequency. Therefore, a high count rate can be obtained with a longer bin time. The gated photon counting approach can also be used to increase the count rate with a short gating interval to provide high detection efficiency and SNR.

Note that the lock-in amplifier extracts the SL signal from an extremely noisy background. The electronic noise is one of the predominant noises for lock-in detection. In addition, lock-in detection is analog signal processing in nature and is more susceptible to noise and signal distortion. Alternatively, digitally detecting the signal through photon counting will greatly improve the electronic noise and the reliability. The gated photon counting can be an advantageous alternative for lock-in detection under the pump-probe scheme.^12^ Notably, the lifetime of the fluorescence dye is in the nanosecond time regime, which allows synchronization of the pump pulses with the probe pulses at half of the repetition frequency (∼38  MHz).^23^ In this way, the highest possible modulation can be achieved and is termed subharmonic synchronization.

## 4 Summary and Future Perspective

In summary, we have successfully established a new pump-probe microscopy technique for the detection of SG and SL signals. In addition, detection of SL allows a much wider dynamic range without saturating the detector and the elimination of the large background, which commonly exists in the SE signal. In this way, fluorescence lifetime images can be obtained with greatly reduced backgrounds.

Finally, and critically, our ultimate goal is to insert the pump-probe microscopy system into a fully digital data acquisition scheme based on photon-counting detection. The pump-probe imaging is equivalent to 2P imaging, which provides optical sectioning capability with high contrast. In particular, our technique is expected to investigate fluorophores and improve SNR in pump-probe microscopy.
